# Hippocampal Homer1 Levels Influence Motivational Behavior in an Operant Conditioning Task

**DOI:** 10.1371/journal.pone.0085975

**Published:** 2014-01-21

**Authors:** Klaus V. Wagner, Alexander S. Häusl, Max L. Pöhlmann, Jakob Hartmann, Christiana Labermaier, Marianne B. Müller, Mathias V. Schmidt

**Affiliations:** Research Group Neurobiology of Stress, Max Planck Institute of Psychiatry, Munich, Bavaria, Germany; University of Minnesota, United States of America

## Abstract

Loss of motivation and learning impairments are commonly accepted core symptoms of psychiatric disorders such as depression and schizophrenia. Reward-motivated learning is dependent on the hippocampal formation but the molecular mechanisms that lead to functional incentive motivation in this brain region are still largely unknown. Recent evidence implicates neurotransmission via metabotropic glutamate receptors and Homer1, their interaction partner in the postsynaptic density, in drug addiction and motivational learning. As previous reports mainly focused on the prefrontal cortex and the nucleus accumbens, we now investigated the role of hippocampal Homer1 in operant reward learning in the present study. We therefore tested either Homer1 knockout mice or mice that overexpress Homer1 in the hippocampus in an operant conditioning paradigm. Our results show that deletion of Homer1 leads to a diverging phenotype that either displays an inability to perform the task or outstanding hyperactivity in both learning and motivational sessions. Due to the apparent bimodal distribution of this phenotype, the overall effect of Homer1 deletion in this paradigm is not significantly altered. Overexpression of hippocampal Homer1 did not lead to a significantly altered learning performance in any stage of the testing paradigm, yet may subtly contribute to emerging motivational deficits. Our results indicate an involvement of Homer1-mediated signaling in the hippocampus in motivation-based learning tasks and encourage further investigations regarding the specific molecular underpinnings of the phenotypes observed in this study. We also suggest to cautiously interpret the results of this and other studies regarding the phenotype following Homer1 manipulations in animals, since their behavioral phenotype appears to be highly diverse. Future studies would benefit from larger group sizes that would allow splitting the experimental groups in responders and non-responders.

## Introduction

Memory deficits and motivational impairments are frequently reported to be associated with the emergence of psychiatric pathologies such as depression [1], [2] and schizophrenia [3]. Motivational behavior has mainly been associated with amygdaloid structures [4], as well as the medial prefrontal cortex [5] and the nucleus accumbens [6]. On the other hand, compelling evidence implicates the hippocampus as a major structure of memory disturbances [7], [8] and reward-motivated learning [9], [10]. As the hippocampal formation is structurally and functionally connected with the amygdala, the prefrontal cortex, and the nucleus accumbens, it can serve as an integrating structure for motivational and memory processes.

In this structural framework, glutamatergic neurotransmission has been shown to be centrally involved in memory formation [11] and reward-seeking behavior, including drug addiction [12]–[14]. Specifically group I metabotropic glutamate receptors (mGluRs) have been shown to interact with scaffolding proteins from the Homer family, which are expressed in the postsynaptic density of glutamatergic neurons. Homer1 has been demonstrated to link group I mGluRs to downstream targets such as inositol triphosphate receptors [15], [16], TRP cation channels [17], and ryanodine receptors [18]. Constitutively expressed Homer1b/c multimers have been shown to mediate ligand-dependent signaling [19], while the shorter splice variant Homer1a, an immediate early gene (IEG) that is induced by neuronal activation [20], can induce ligand-independent signaling and is thought to act as a dominant negative to the constitutively expressed isoform [21].

A number of clinical and preclinical reports have implicated Homer1 in the pathophysiology of depression [22], schizophrenia [23], [24] and addiction [25], [26]. In these studies, the role of Homer1 in reward-associated behavior has also been explored. In rodent studies, the Homer1/mGluR5 signaling pathway has previously been shown to be involved in memory formation and cognition in the prefrontal cortex [27] and the hippocampus [28]–[30]. Furthermore, mGluR5/Homer1 interactions have been shown to mediate stress-induced alterations in memory formation of both fear conditioning [31] and spatial information [32] in mice. However, the role of hippocampal Homer1/mGluR5 in operant reward learning and motivation, which are central aspects for mood disorders, is still largely unclear.

In the current study, we therefore aimed to further elucidate the role of hippocampal Homer1 in operant reward learning by testing Homer1 knockout mice as well as mice that overexpress the constitutively expressed Homer1b/c isoform in the hippocampus in an operant conditioning paradigm. We hypothesized that a deletion of Homer1 leads to a reduction of incentive motivation [25], while overexpression of Homer1b/c in the hippocampus should improve memory formation and thereby may help to improve the performance in the operant conditioning task.

## Materials and Methods

### Animals

Conventional Homer1KO mice were bred from heterozygous breeding pairs on a C57BL/6N background in the animal facilities of the Max Planck Institute of Psychiatry in Munich, Germany. Generation and genotyping of Homer1KO mice was reported previously [17]. Homer1 knockout resulted in complete loss of protein expression and was verified by PCR. For the Homer1b/c overexpression experiment, male C57BL/6N mice (Charles River Laboratories, Maastricht, the Netherlands) at the age of 10 weeks were used. All mice were held under standard conditions (12L∶12D light cycle, lights on at 08:00 AM, temperature 23±2°C) and were single housed and acclimated to the experimental room for 2 weeks before the beginning of the experiments. Tap water was available *ad libitum* during the whole experiment. Food (Altromin 1324, Altromin GmbH, Germany) was available *ad libitum* until start of the food restriction period. All experiments were performed in the animal facilities of the Max Planck Institute of Psychiatry in Munich, Germany. The experiments were carried out in accordance with the European Communities' Council Directive 2010/63/EU. All efforts were made to minimize animal suffering during the experiments. The protocols were approved by the committee for the Care and Use of Laboratory animals of the Government of Upper Bavaria, Germany.

### Experimental design

#### Experiment 1

Adult male Homer1 knockout (KO) mice or wild type (WT) littermate controls (12 to 14 weeks of age, n = 9–10 per group) were tested in the operant conditioning paradigm.

#### Experiment 2

Adult male C57Bl/6 mice received intra-hippocampal injections of a Homer1b/c overexpression vector (n = 10) or an empty control virus (n = 10) and were tested in the operant conditioning paradigm 4 weeks later.

### Operant conditioning

After one week of single housing in the experimental room, the average daily food intake was measured for 5 days for each individual. Based on the mean of these 5 measurements, food was subsequently restricted to 80% of the daily intake until the end of the experiment (Figure S1). The animals received the reduced daily portion of food pellets at 2:00 PM. This mild caloric restriction has been shown to have no negative consequences for the physiological wellbeing of the animals but promotes incentive motivation in the operant conditioning task [24]. After 7 days of food restriction without further experimental interference, animals were introduced to the operant conditioning chamber (Bioseb, France) for 5 days. In each 30 min trial, mice received a sucrose reward (Bio-serv, NJ, USA) every 45 s, which was always paired with a 3 s light and sound (5000 Hz) stimulus. In preliminary experiments, the paired audio-visual sensory stimulus emerged as most effective. Reward delivery and stimuli were operated with commercially available software (Packwin V2.0.01; Panlab, Spain).

The training stage consisted of a fixed ratio/variable ratio (FR/VR) protocol, in which the experimental animals received a reward after a single lever press for the first ten presses (FR1) followed by 1–3 lever presses to receive a reward (VR1-3). The 30 min training trial was performed in bouts of 5 consecutive daily trials per week, until 75% of mice in the respective control group (WT or Empty) received at least 10 rewards. In the first experiment, this was the case after 15 training trials. Here, 2 WT and 5 KO animals did not pass the cut-off criterion and were not tested in the progressive ratio task. In the second experiment, 10 training trials were performed. 2 Empty and 4 Homer1b/c OE animals did not pass the cut-off criterion and were excluded from subsequent testing.

Mice that passed the training stage were tested in a progressive ratio (PR) task for 120 min to test the animals for motivation in the previously acquired operant conditioning task. Since the experimental groups in this task are significantly smaller than the initial sample size in both experiments, the results of the PR task have not been analyzed statistically but rather serve as a descriptive observation and extension of the previously observed phenotypes in the training sessions. The reward progression for the PR task is outlined in Table S1 in File S1.

The whole time course of the experiments, including surgery, recovery, food restriction and habituation is shown in Figure S1. All operant conditioning trials were performed between 8:00 AM and 12:00 PM. After experiment 2, all animals were deeply anesthetized with ketamine/rompun and perfused intracardially with 4% paraformaldehyde. Brains were removed, postfixed overnight in 4% paraformaldehyde following overnight incubation in 30% sucrose solution at 4°C, and then stored at −80°C until further processing for immunohistochemistry as described below.

### Locomotion

Locomotion was recorded and analyzed over the course of the progressive ratio task, using an automated videotracking system (Anymaze 4.20; Stoelting) as described before [32].

### Viral overexpression of Homer1

Viral overexpression of Homer1b/c was performed as described previously [33]. We used an adeno-associated bicistronic AAV1/2 vector (GeneDetect, New Zealand) containing the CAG-Homer1-IRES-EGFP-WPRE-BGH-polyA expression cassette (containing coding sequence of Homer1 NCBI CCDS ID CCDS36745). For the control group, we used the same vector construct expressing only EGFP. Virus production, amplification, and purification were performed by Genedetect. Mice were anesthetized with isoflurane, and 0.5 µL of either AAV-Homer1 or AAV-EGFP (titres: 1.2×10^12^ genomic particles/mL) were bilaterally injected in the dorsal hippocampus at 0.06 µL/min by glass capillaries with tip resistance of 2–4 MΩ in a stereotactic apparatus. The following coordinates were used: 1.9 mm posterior to bregma, 1.3 mm lateral from midline, and 1.3/1.8 mm below the surface of the skull, targeting the CA1 and dentate gyrus (DG) region of the dorsal hippocampus. After surgery, mice were treated for 5 d with Metacam via drinking water. The habituation phase of the operant conditioning paradigm started 4 weeks after virus injection. Quantification and verification of Homer1b/c overexpression were confirmed by *in situ* hybridization and immunofluorescence as described previously [32]. Animals that were not infected bilaterally in both the CA1 and DG region were excluded from the analysis (n = 1). One mouse (Empty group) died in the recovery phase after the surgery, before the experiment started.

### In Situ Hybridization and Immunohistochemistry

For *in situ* hybridization, frozen brains were coronally sectioned in a cryostat microtome at 18 µm and kept at −80°C. In situ hybridization using a ^35^S UTP-labeled ribonucleotide probe for Homer1b/c (Forward primer: AACACTGGGAGGCTGAGCTA; Reverse primer: TACTGCGGAAAGCCTCTTGT) was performed as described previously [34]. For fluorescence immunohistochemistry, serial coronal sections were cut at 30 µm thickness. Double-labeling immunofluorescence (rabbit anti-Homer1, 1∶1000, Synaptic Systems; goat anti-GFP, 1∶500, Abcam) was performed on free-floating sections (n = 3 per mouse) as described previously [35].

### Statistical Analysis

The data presented is shown as means ± standard error of the mean, analyzed by the commercially available software SPSS 16.0. Repeated measures ANOVA with time as within-subjects factor and genotype/AAV type as between-subjects factor or Chi Square analysis were used for body weight, habituation and training stage analysis. For the food intake, data were analyzed with student's *t*-test. Correlations between lever presses and locomotion were analyzed with the Pearson product-moment test. A nominal level of significance p<0.05 was accepted.

## Results

### Experiment 1

Homer1KO mice displayed a significantly reduced body weight already at the beginning of the experiment (WT: 27.76±0.62 g, KO: 23.55±0.85 g; p<0.001), but no difference was detected in the daily food intake (WT: 3.58±0.03 g, KO: 3.72±0.11 g; p = 0.29). Over the course of the operant conditioning paradigm, food restriction resulted in a body weight loss in both groups, independent of the genotype of the animals (time effect: F_2.072_ = 11.365, p<0.001) (Table S2 in File S1) Also, the reduced body weight of Homer1KO mice compared to their WT littermates was present during the whole experimental period (F_1,10_ = 21.312; p<0.001).

**Figure 1:**
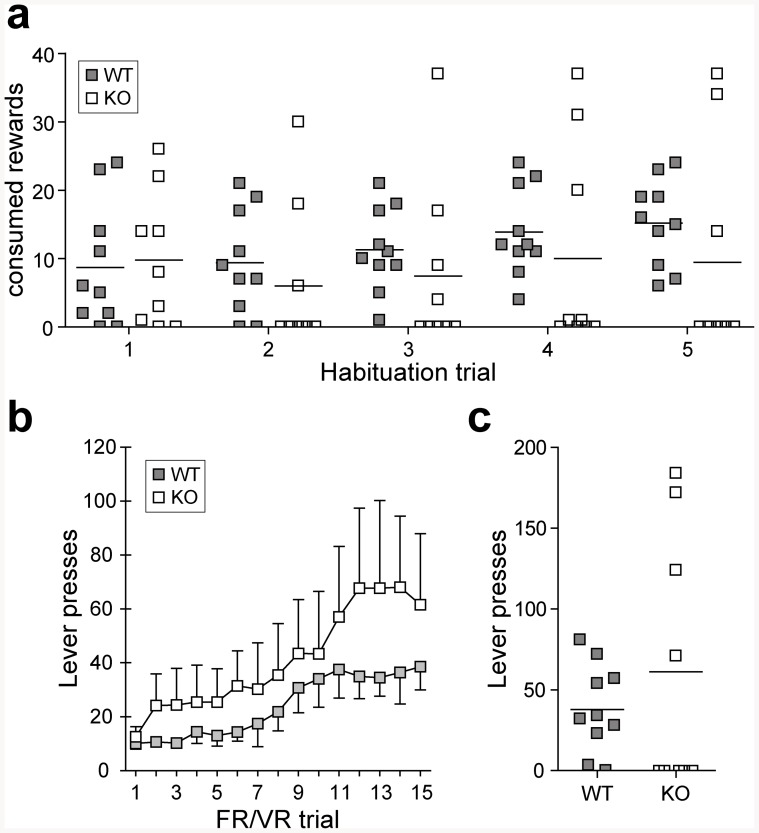
Training performance of Homer1KO mice. (**a**) In 5 habituation trials, all wild type (WT) mice show growing interest in the presented reward. Most of the Homer1KO mice, however, do not consume the sucrose pellets. (**b**) The learning curve in the fixed ratio/variable ratio (FR/VR) stage is slightly, but not significantly higher in Homer1KO mice compared to WT animals. This is due to the above-average performance of a subset of Homer1KO mice that already showed a response to the reward in the habituation phase, while the greater part of the Homer1KO animals show a below-average performance, thereby largely increasing the variance of the sample. (**c**) FR/VR results of training trial 15. A strong bimodal distribution of the Homer1KO group becomes apparent, consequently resulting in no significant difference when compared to WT animals.

In the habituation phase, repeated measures ANOVA reveals a time effect (F_2.591_ = 3.190; p<0.05) but no time × genotype interaction in the number of consumed rewards, indicating that all animals showed increased interest in the sucrose pellets over time (Figure 1a). Yet, it has to be noted that 6 out of 9 Homer1KO mice did not consume any reward in the fifth habituation trial. The same animals did not express interest in the reward in previous habituation trials, while WT mice displayed a normally distributed interest in the reward. This genotype difference becomes significant over several trials when analyzed by a Chi Square test (Trial 1: p = 0.667, Trial 2: p = 0.055, Trials 3 to 5: p<0.05).

Over the course of the FR/VR training period, WT animals displayed a normal learning behavior with a stable lever press response after 15 training trials (Figure 1b). The mean of Homer1KO mice lever press responses also increased steadily, which is reflected in a significant repeated measures ANOVA main time effect (F_2.175_ = 5.340; p<0.01) without significant time × genotype interaction. A more detailed analysis of the Homer1KO dataset revealed that 5 of 9 subjects pressed the lever less than 5 times and did not consume any presented reward in the majority of the training trials. On the other hand, those animals that already showed high interest in the reward during habituation also performed above average in the training stages, thereby largely increasing the variance in the Homer1KO group. After training trial 15, 8 of 10 WT mice passed the cut-off criterion of 10 lever presses, while only 4 of 9 KO animals received more than 10 rewards (Figure 1c). The results of the PR task can be found in Figure S2.

### Experiment 2

**Figure 2:**
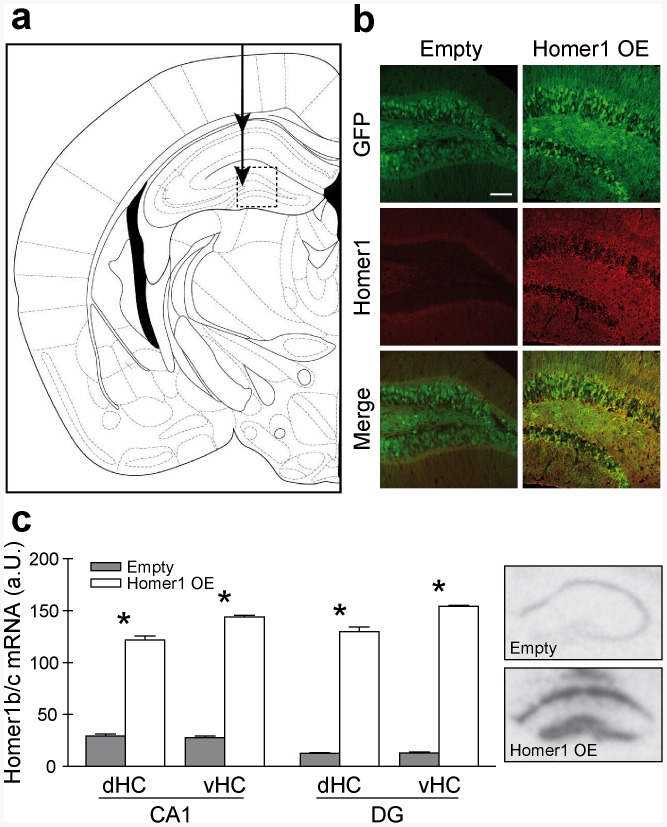
Confirmation and quantification of viral overexpression. (**a**) Schematic of the injection site of the virus in the CA1 and the dentate gyrus (DG) of the dorsal hippocampus. The dotted square indicates the approximate area of visualization in Panel b. (**b**) Visualization of Homer1b/c expression in the hippocampal DG region 8 weeks after injection of control (Left panels) or Homer1b/c-expressing virus (Right panels) (Scale: 100 µm). (**c**) Homer1b/c mRNA levels in the hippocampus. Infection with the viral construct induced a robust increase in both CA1 and DG mRNA levels in the dorsal (dHC) and ventral (vHC) part of the hippocampus. Pictures show representative autoradiographs of Homer1b/c mRNA levels in the dorsal hippocampus of empty and Homer1 OE animals. * Significant from Empty virus, p<0.05.

Successful targeting (Figure 2a) and overexpression of Homer1b/c was validated by immunohistochemistry (Figure 2b) and quantified by means of in situ hybridization (Figure 2c). Both in the dorsal (dHC) and the ventral hippocampus (vHC), we detected a significant increase in Homer1b/c mRNA levels in CA1 (dHC: T_16_ = −22.728, p<0.001; vHC: T_16_ = −48.992, p<0.001) and DG regions (dHC: T_16_ = −25.885, p<0.001; vHC: T_16_ = −101.802, p<0.001). Viral spread was analogous to our previous study with this viral construct [32].

While overexpression of Homer1b/c did not lead to a significant change in body weight during the experiment, food restriction led to a body weight reduction in both groups over time (time effect: F_1,326_ = 21.312; p<0.001) (Table S3 in File S1). The daily food intake did not differ between the groups (Empty: 3.32±0.1 g, Homer1 OE: 3.55±0.08 g; p = 0.1).

**Figure 3:**
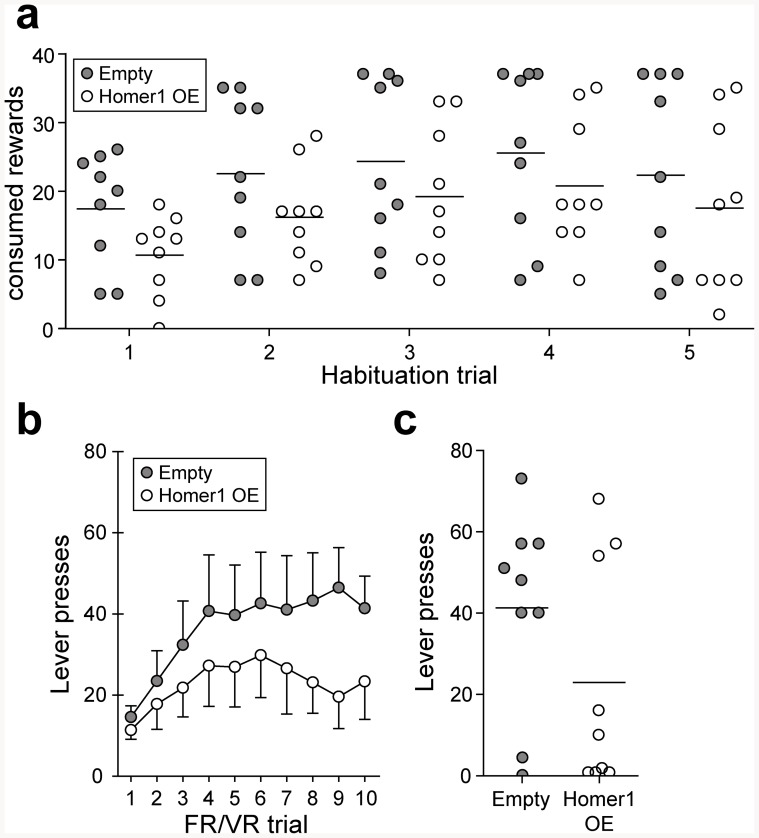
Training performance of Homer1 OE mice. (**a**) During habituation, both groups quickly recognized and consumed the presented rewards. No differences between the treatments were detected. (**b**) During the training stage, both Empty and Homer1 OE animals learned to associate lever presses with the reception of a reward. Although Homer1 OE mice appear to show less motivational behavior, repeated measures ANOVA did not reveal a significant time × AAV interaction. (**c**) Fixed ratio/variable ratio results of training trial 10. No difference in lever press activity was evident between the experimental groups.

Both Empty and Homer1b/c OE animals showed increasing interest in the presented reward over the course of the habituation phase (time effect: F_1.584_ = 9.170; p<0.01) with no time ×AAV effect (F_1.584_ = 0.166; p = 0.798) or Chi Square significance between the AAV types (Figure 3a). Overexpression of Homer1b/c did not have an effect on the consumed reward number. Note that during this stage, a maximum of 37 rewards could be consumed. This limit was reached by three Empty animals in both the 4^th^ and the 5^th^ habituation trial.

During the training trials, both experimental groups showed a time-dependent increase in lever presses to receive rewards (time effect: F_2.977_ = 4.087; p<0.05), without a significant effect of Homer1b/c overexpression or interaction effects (Figure 3b). After training trial 10, 7 out of 9 Empty animals received more than 10 rewards, thereby passing the cut-off criterion. In the Homer1 OE group, only 5 out of 9 animals exceeded the amount of lever presses to pass the criterion (Figure 3c). The results of the PR task can be found in Figure S3.

## Discussion

In the current study, we provide first indications that hippocampal Homer1 may be involved in operant reward learning. In an extensive operant conditioning paradigm, we tested both Homer1KO and Homer1 OE mice with respect to their learning and motivational behavior. In the Homer1KO animals, two distinct subgroups emerged: mice that displayed high motivation and activity and animals that did not perform at all in the operant conditioning task. Overexpression of Homer1b/c in the hippocampus did not affect the basic interest in sucrose rewards. These results extend the current knowledge that Homer1 signaling plays a crucial role in functional incentive motivation specifically in the hippocampus, and further suggests that Homer1 may be a relevant target for the treatment of psychiatric disorders such as depression or schizophrenia.

The complex behavioral phenotype of Homer1KO mice has previously been associated with learning and memory deficits and motivational impairments [24]. Szumlinski and colleagues could show that Homer1KO animals display less motivation to obtain a sucrose reward. Pronounced hyperactivity indicated by increased locomotion in a novel environment [24] and enhanced activity in the rest cycle [29], has also been reported in these mice, which was confirmed by observations made in our group (unpublished data). In our study, we observed that most of the Homer1KO animals did not express any interest in the presented reward, while others displayed an abnormally high activity, reflected by excessive retrieval and consummation of the rewards, yet these effects failed to reach significance due to the reduced animal number in each subpopulation. This bimodal distribution is apparent over the course of the various tasks and makes data interpretation difficult, since the results may not necessarily represent learning as much as distinct hyperactivity of a subset of KO animals. Although learning deficits have frequently been reported in the context of Homer1 deletion [25], [27], it is likely that these impairments play a secondary role to the observed inactive phenotype. Since the animals were not required to learn a task before acquiring a reward in the habituation phase, we suggest that the majority of Homer1KO mice initially showed indifference or even a degree of aversion towards the reward. Subsequently these mice apparently did not regard the sugar pellets as a sufficient stimulus to develop a motivational drive in the successive stages of the experiment and therefore did not comply with the reward-stimulus paradigm presented in the following weeks. Such findings indicate that deletion of Homer1 may have opposing effects on individuals in the same task, most likely depending on environmental factors that have yet to be elucidated. Nonetheless, these preliminary findings need to be further substantiated in larger samples. Deletion of Homer1 is already strongly affecting the animals in early life stages, resulting in high mortality for yet unknown reasons and potential detrimental effects on the surviving pups. This has consistently been observed in our breeding colony and other labs [36], and may likely contribute to individual differences due to a strong genotype × environment interaction. The food restriction used here may be another factor leading to individual differences in the measured behaviors. Interestingly, food-sated Homer1KO mice have indeed been reported to show altered performance in reward-seeking tasks when compared to food deprived animals [24]. In our study, Homer1KO animals presented significantly lower body weight from the onset of the experiment, a phenotype that has not been previously reported [25], [29]. A reduced absolute weight may alter the severity of the food restriction and therefore confound the motivational alterations in comparison to the WT control group. This is especially intriguing since Homer1 is also deleted in cortical regions, which may interfere with appetite and hunger perception. Nonetheless, there was no difference in the actual amount of consumed food between WT and KO animals, which gives rise to the hypothesis that a total deletion of Homer1 may also have effects in the periphery that alter metabolic processes or the physiology of these animals with respect to energy balance.

To specifically address the question of whether hippocampal Homer1 expression has an impact on operant reward learning, we overexpressed Homer1b/c by viral transfection and exposed these animals to the same operant conditioning paradigm. We did not observe a significant basal difference in the motivation for sucrose reward during the habituation phase. This also translates into the training stage, where no significant interactions could be found. However, a large subgroup of Homer1 OE mice did not reach the cut-off criterion after the training stage, hinting at problems with operant conditioning memory processes in these animals. This is surprising, since Homer1b/c has frequently been linked to improved memory processing [19], [27], [28]. Confounding effects of general activity are not likely, since these animals did not show altered locomotion in the behavioral setup. Additionally, previous phenotyping of animals that overexpress Homer1 in the hippocampus did not reveal basal effects on behavioral core parameters [32]. A possible underlying mechanism for these effects may be caused by the imbalance between overexpressed Homer1b/c and the IEG Homer1a, which has been shown to be critically involved in memory formation [37]. A recent study has shown that Homer1a is required for fear conditioning and furthermore that fear conditioning induces the upregulation of this gene [38]. Hernandez and colleagues reported an increase in Homer1a mRNA levels in rats after an instrumental learning task, further supporting the importance of this IEG in operant conditioning tasks [39]. The elevated levels of Homer1b/c in the hippocampus of Homer1 OE mice may be causal for the induction of Homer1a not being sufficient to trigger the downstream pathways that in turn stimulate motivational behavior. Interestingly, both KO and OE mice showed problems in reaching the cut-off criterion, suggesting that general modulation or dysbalance of the Homer1 signaling system, i.e. the ratio between Homer1b/c and Homer1a levels, may be detrimental to learning. Therefore, further studies that specifically investigate the role of Homer1a, e.g. by pharmacological induction, in the current model may help to understand the significance of hippocampal Homer1a/Homer1b/c interplay in operant conditioning learning and motivational behavior.

A major limitation of this study emerges from the relatively small number of animals in each experimental group. In particular, the bimodal distribution of the Homer1KO mice complicates the data interpretation. We therefore refrained from analyzing the results of the PR tasks, since the statistical power of the sample size is too low. Future studies should consider larger group compositions to further investigate possible underlying mechanisms of this diverging phenotype. Concerning the Homer1 OE animals, follow-up studies need to address the question as to whether loss of reward motivation is indeed linked to the reduced ability to learn the operant conditioning task. Also, the overexpression of Homer 1b/c exclusively was limited to the hippocampus. Conversely, the KO mice suffered from complete loss of all Homer1 subtypes across all brain regions and a specific knockdown in the hippocampus may provide additional insights into the specific role of Homer1 loss. Furthermore, the performances of the control groups in both experiments differ, yet this may be attributed to the different origin and of the animals used. However, this has to be kept in mind when directly comparing the different phenotypes of both experiments. A more detailed molecular analysis of hippocampal Homer1 interaction partners, especially in Homer1KO mice, may lead to further insight in this respect.

Taken together, we provide indications that hippocampal Homer1 is involved in the acquisition of an operant conditioning paradigm, with a potential decrease of motivational behavior in mice that overexpress Homer1b/c in the hippocampus. Additionally we detected hyperactive behavior in a subpopulation of Homer1KO mice that has not been previously described, strongly suggesting the need to further investigate this mouse model, on both a behavioral and molecular level. The results presented in this study provide further evidence that alterations in signaling pathways, specifically Homer1, may contribute to the emergence of motivational and learning deficits.

## Supporting Information

Figure S1 **Experimental design overview.** (a) In experiment 1, Homer1KO mice were trained in the FR/VR protocol for 3 weeks until the PR test session was performed. (b) In experiment 2, Homer1 OE animals were allowed to recover for 3 weeks until food restriction commenced. FR/VR training lasted for 2 weeks. FR/VR: Fixed ratio/Variable ratio; PR: Progressive ratio.
(TIF)

Figure S2 **Progressive ratio (PR) performance of Homer1KO mice.** (**a**) In the PR task, the remaining Homer1KO animals showed a high amount of lever press activity. We did not statistically analyze the data, as there were only 4 animals left in the KO subgroup. The arrows indicate the datapoints that are plotted in panel c. (**b**) locomotion was also neither different from the WT group nor was it correlated to the lever presses in the PR task. (**c**) Representative cumulative distribution of PR lever presses. Dispensed rewards are marked as triangles. The Homer1KO mouse (black line) shows high performance over the course of 120 min, thereby receiving constant rewards. In contrast, the wild type mouse (grey line) shows less operant responses once rewards are obtained more and more slowly.
(TIF)

Figure S3 **Progressive ratio (PR) performance of Homer1 OE mice.** (**a**) Homer1 OE mice appear to press the lever less frequent compared to Empty animals in the PR task. We did not statistically analyze the data, as there were only 5 animals left in the OE subgroup The arrows indicate the datapoints that are plotted in panel c. (**b**) Locomotion in the PR task. Overexpression of Homer1b/c does not lead to a general increase in locomotion. (**c**) Representative cumulative distribution of PR lever presses. Dispensed rewards are marked as triangles. The mouse infected with empty virus (black line) shows high activity up to 60 min into the PR stage, followed by a decreased lever press frequency. This activity decrease appears earlier in the Homer1OE animal (grey line), which translates into a reduced overall activity over the course of 120 min.
(TIF)

File S1 **Tables S1–S3.** Table S1. Progressive ratio reward overview. The middle column shows the required amount of lever presses to achieve the next reward. The right column shows the cumulated total lever presses required to receive the respective number of rewards given in the left column. Table S2. Body weight progression in experiment 1. Repeated measures ANOVA revealed an effect of time (F_5, 6_ = 25.091; p<0.001) and a significant between subject genotype effect (F_1, 10_ = 21.312; p<0.001). Homer1KO animals were significantly lighter over the course of the experiment than their WT littermates, while both groups were affected by the food restriction (colored in grey). Table S3. Body weight progression in experiment 2. Repeated measures ANOVA revealed an effect of time (F_5, 6_ = 30.964; p<0.001) but no effect of the Virus on body weight progression. Both groups lost weight in response to the food restriction (colored in grey).
(DOC)
