# Supplementary figures and images for: Hippocampal Homer1 Levels Influence Motivational Behavior in an Operant Conditioning Task
Source: PLoS One. 2014 Jan 21;9(1):e85975. doi: 10.1371/journal.pone.0085975 (PMC3897610; doi:10.1371/journal.pone.0085975)

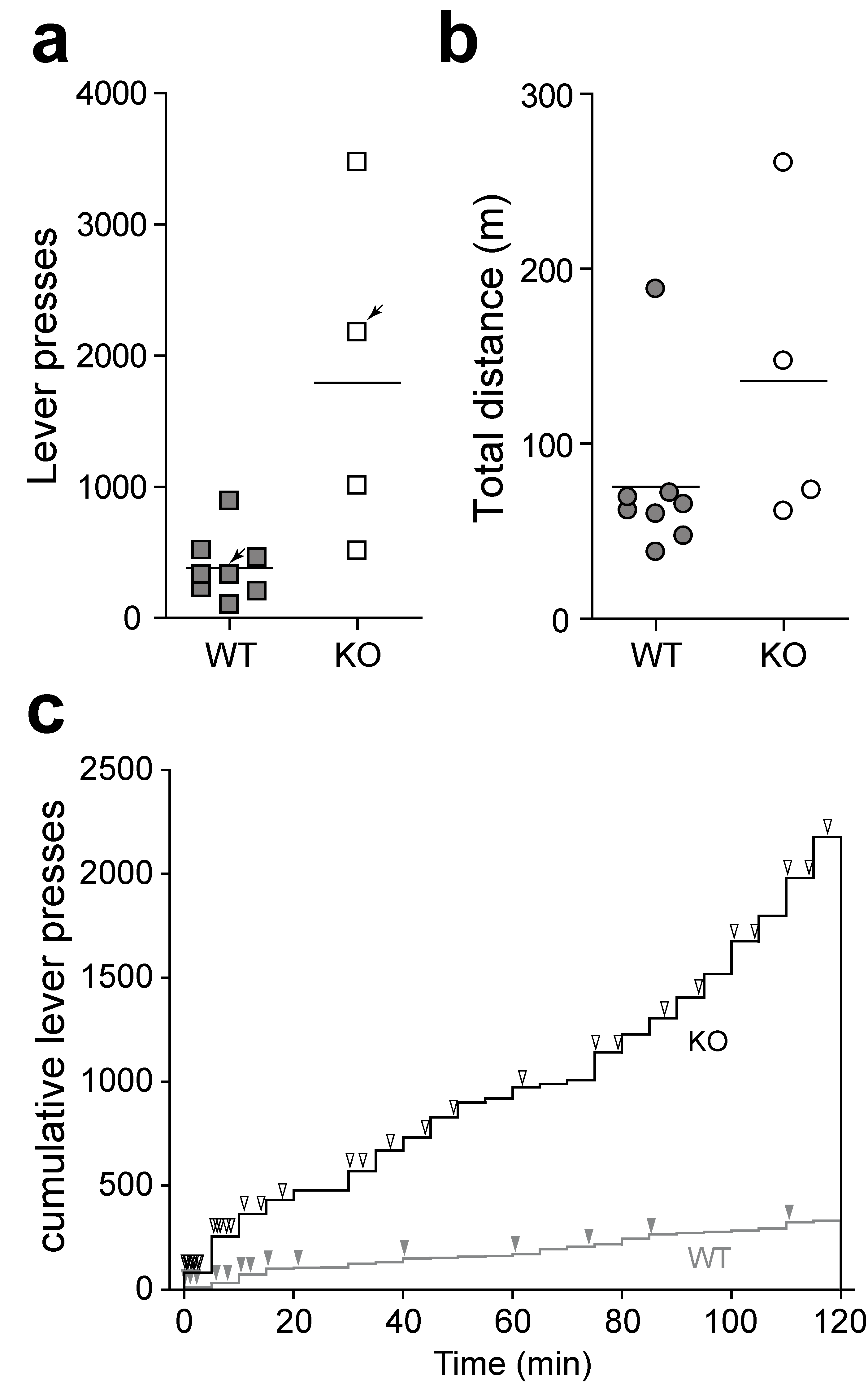

Supplement: Figure S2 — Progressive ratio (PR) performance of Homer1KO mice. (a) In the PR task, the remaining Homer1KO animals showed a high amount of lever press activity. We did not statistically analyze the data, as there were only 4 animals left in the KO subgroup. The arrows indicate the datapoints that are plotted in panel c. (b) locomotion was also neither different from the WT group nor was it correlated to the lever presses in the PR task. (c) Representative cumulative distribution of PR lever presses. Dispensed rewards are marked as triangles. The Homer1KO mouse (black line) shows high performance over the course of 120 min, thereby receiving constant rewards. In contrast, the wild type mouse (grey line) shows less operant responses once rewards are obtained more and more slowly. (TIF) [file pone.0085975.s002.tif]

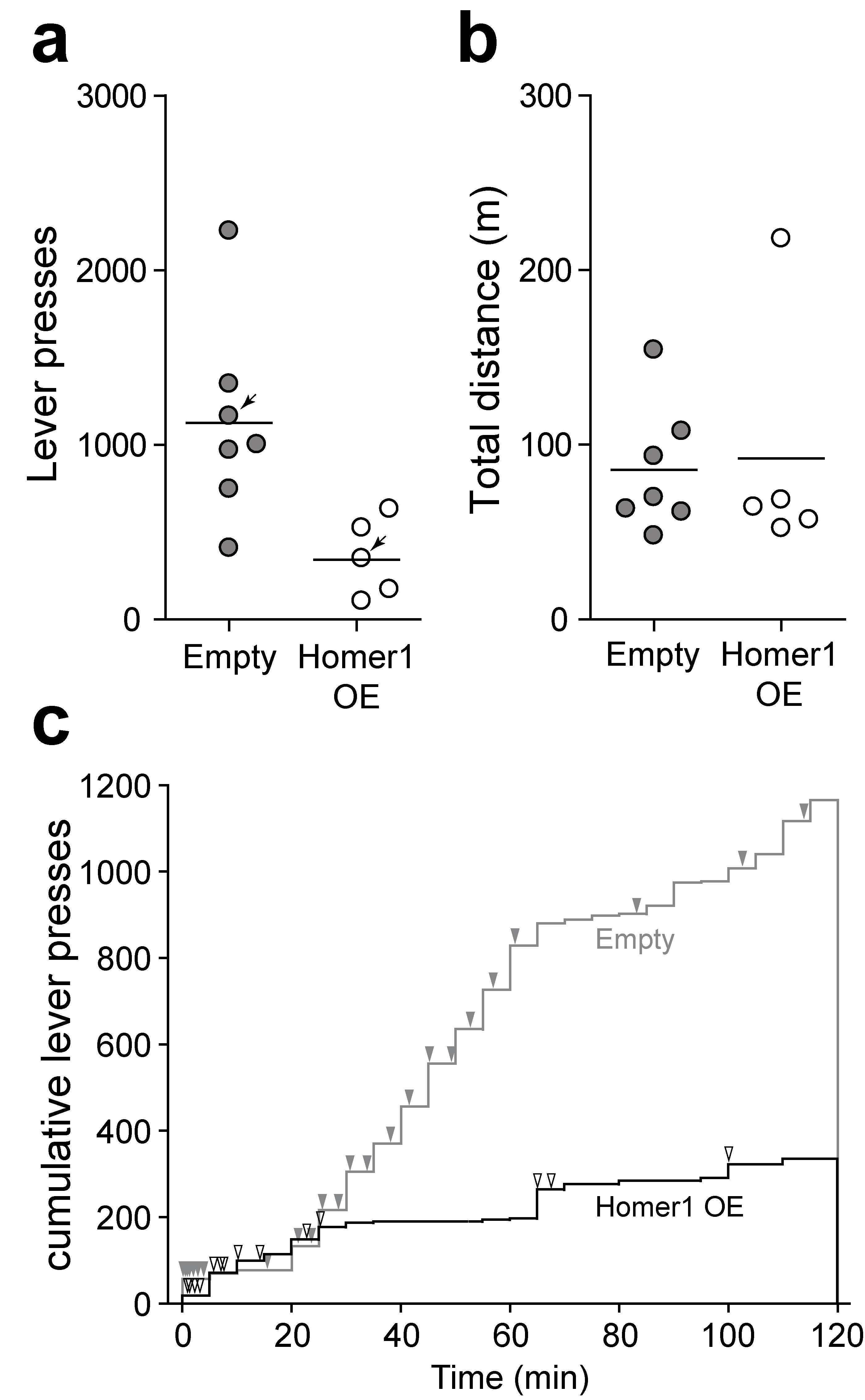

Supplement: Figure S3 — Progressive ratio (PR) performance of Homer1 OE mice. (a) Homer1 OE mice appear to press the lever less frequent compared to Empty animals in the PR task. We did not statistically analyze the data, as there were only 5 animals left in the OE subgroup The arrows indicate the datapoints that are plotted in panel c. (b) Locomotion in the PR task. Overexpression of Homer1b/c does not lead to a general increase in locomotion. (c) Representative cumulative distribution of PR lever presses. Dispensed rewards are marked as triangles. The mouse infected with empty virus (black line) shows high activity up to 60 min into the PR stage, followed by a decreased lever press frequency. This activity decrease appears earlier in the Homer1OE animal (grey line), which translates into a reduced overall activity over the course of 120 min. (TIF) [file pone.0085975.s003.tif]
