# Supplementary material for: Hippocampal Homer1 Levels Influence Motivational Behavior in an Operant Conditioning Task
Source: PLoS One. 2014 Jan 21;9(1):e85975. doi: 10.1371/journal.pone.0085975 (PMC3897610; doi:10.1371/journal.pone.0085975)
Supplement: File S1 — Tables S1–S3. Table S1. Progressive ratio reward overview. The middle column shows the required amount of lever presses to achieve the next reward. The right column shows the cumulated total lever presses required to receive the respective number of rewards given in the left column. Table S2. Body weight progression in experiment 1. Repeated measures ANOVA revealed an effect of time (F5, 6 = 25.091; p<0.001) and a significant between subject genotype effect (F1, 10 = 21.312; p<0.001). Homer1KO animals were significantly lighter over the course of the experiment than their WT littermates, while both groups were affected by the food restriction (colored in grey). Table S3. Body weight progression in experiment 2. Repeated measures ANOVA revealed an effect of time (F5, 6 = 30.964; p<0.001) but no effect of the Virus on body weight progression. Both groups lost weight in response to the food restriction (colored in grey). (DOC) [file pone.0085975.s004.doc]

**Supporting Material**

| **Reward No.** | Lever presses for next reward | Total lever presses |  | **Reward No.** | Lever presses for next reward | Total lever presses |
| --- | --- | --- | --- | --- | --- | --- |
| **1** | 1 | 1 |  | **27** | 132 | 1486 |
| **2** | 2 | 3 |  | **28** | 139 | 1625 |
| **3** | 4 | 7 |  | **29** | 147 | 1772 |
| **4** | 7 | 14 |  | **30** | 155 | 1927 |
| **5** | 10 | 24 |  | **31** | 163 | 2090 |
| **6** | 13 | 37 |  | **32** | 171 | 2261 |
| **7** | 17 | 54 |  | **33** | 179 | 2440 |
| **8** | 21 | 75 |  | **34** | 187 | 2627 |
| **9** | 25 | 100 |  | **35** | 195 | 2822 |
| **10** | 29 | 129 |  | **36** | 203 | 3025 |
| **11** | 34 | 163 |  | **37** | 212 | 3237 |
| **12** | 39 | 202 |  | **38** | 221 | 3458 |
| **13** | 44 | 246 |  | **39** | 230 | 3688 |
| **14** | 49 | 295 |  | **40** | 239 | 3927 |
| **15** | 54 | 349 |  | **41** | 248 | 4175 |
| **16** | 60 | 409 |  | **42** | 257 | 4432 |
| **17** | 66 | 475 |  | **43** | 266 | 4698 |
| **18** | 72 | 547 |  | **44** | 275 | 4973 |
| **19** | 78 | 625 |  | **45** | 284 | 5257 |
| **20** | 84 | 709 |  | **46** | 294 | 5551 |
| **21** | 90 | 799 |  | **47** | 304 | 5855 |
| **22** | 97 | 896 |  | **48** | 314 | 6169 |
| **23** | 104 | 1000 |  | **49** | 324 | 6493 |
| **24** | 111 | 1111 |  | **50** | 334 | 6827 |
| **25** | 118 | 1229 |  | **51** | 344 | 7171 |
| **26** | 125 | 1354 |  | **52** | 354 | 7525 |

Supporting table 1: Progressive ratio reward overview. The middle column shows the required amount of lever presses to achieve the next reward. The right column shows the cumulated total lever presses required to receive the respective number of rewards given in the left column.

| Genotype | Food intake measurement | Food restriction | Habituation | FR/VR training 1 | FR/VR training 2 | FR/VR training 3 | PR test |
| --- | --- | --- | --- | --- | --- | --- | --- |
| WT | 27.76 ± 0.62 | 26.42 ± 0.67 | 25.53 ± 0.55 | 25.32 ± 0.59 | 25.74 ± 0.56 | 25.72 ± 0.56 | 25.14 ± 0.65 |
| KO | 23.55 ± 0.85 | 21.41 ± 0.92 | 21.63 ± 0.71 | 21.02 ± 0.75 | 21.62 ± 0.71 | 21.39 ± 0.74 | 21.06 ± 0.72 |

Supporting table 2: body weight progression in experiment 1. Repeated measures ANOVA revealed an effect of time (F_5, 6_ = 25.091; p < 0.001) and a significant between subject genotype effect (F_1, 10_ = 21.312; p < 0.001). Homer1KO animals were significantly lighter over the course of the experiment than their WT littermates, while both groups were affected by the food restriction (colored in grey)

| AAV type | Food intake measurement | Food restriction | Habituation | FR/VR training 1 | FR/VR training 2 |  | PR test |
| --- | --- | --- | --- | --- | --- | --- | --- |
| Empty | 30.46 ± 1.04 | 27.81 ± 0.95 | 27.71 ± 0.78 | 28.44 ± 0.71 | 26.34 ± 0.60 |  | 26.77 ± 0.62 |
| Homer1 OE | 31.62 ± 0.80 | 29.13 ± 0.65 | 29.23 ± 0.62 | 29.62 ± 0.45 | 27.28 ± 0.21 |  | 27.06 ± 0.14 |

Supporting table 3: body weight progression in experiment 2. Repeated measures ANOVA revealed an effect of time (F_5, 6_ = 30.964; p < 0.001) but no effect of the Virus on body weight progression. Both groups lost weight in response to the food restriction (colored in grey).
